# Therapeutic Potential of Targeting Periostin in the Treatment of Graves’ Orbitopathy

**DOI:** 10.3389/fendo.2022.900791

**Published:** 2022-05-30

**Authors:** Sun Young Jang, Jinjoo Kim, Jung Tak Park, Catherine Y. Liu, Bobby S. Korn, Don O. Kikkawa, Eun Jig Lee, Jin Sook Yoon

**Affiliations:** ^1^ Department of Ophthalmology, Soonchunhyang University Bucheon Hospital, Soonchunhyang University College of Medicine, Bucheon, South Korea; ^2^ Department of Ophthalmology, Severance Hospital, Institute of Vision Research, Yonsei University College of Medicine, Seoul, South Korea; ^3^ Department of Internal Medicine, College of Medicine, Institute of Kidney Disease Research, Yonsei University, Seoul, South Korea; ^4^ Division of Oculofacial Plastic and Reconstructive Surgery, University of California, San Diego, La Jolla, CA, United States; ^5^ Division of Endocrinology, Department of Internal Medicine, Yonsei University College of Medicine, Seoul, South Korea

**Keywords:** periostin, Graves orbitopathy, fibroblasts, inflammation, fibrosis, adipogenesis

## Abstract

Periostin is a matricellular protein that is ubiquitously expressed in normal human tissues and is involved in pathologic mechanism of chronic inflammatory and fibrotic disease. In this study we investigate periostin in the pathogenesis of Graves’ orbitopathy (GO) using human orbital adipose tissue obtained from surgery and primary cultured orbital fibroblasts *in vitro*. POSTN (gene encoding periostin) expression in Graves’ orbital tissues and healthy control tissues was studied, and the role of periostin in GO pathologic mechanism was examined through small-interfering RNA (siRNA)-mediated silencing. POSTN gene expression was significantly higher in Graves’ orbital tissues than healthy control tissues in real-time PCR results, and immunohistochemical staining revealed higher expression of periostin in Graves’ orbital tissues than normal tissues. Silencing periostin using siRNA transfection significantly attenuated TGF-β-induced profibrotic protein production and phosphorylated p38 and SMAD protein production. Knockdown of periostin inhibited interleukin-1 β -induced proinflammatory cytokines production as well as phosphorylation of NF-κB and Ak signaling protein. Adipocyte differentiation was also suppressed in periostin-targeting siRNA transfected GO cells. We hypothesize that periostin contributes to the pathogenic process of inflammation, fibrosis and adipogenesis of GO. Our study provides *in vitro* evidence that periostin may be a novel potential therapeutic target for the treatment of GO.

## Introduction

Graves’ orbitopathy (GO) is an autoimmune disease of the orbital soft tissues, which occurs in patients with Graves’ disease (1). Thyroid stimulating hormone receptor (TSHR) is an autoantigen shared by the orbit and the thyroid gland (2). It is potentially blinding and is initiated by self-reactive T cells activated against autoantigens on orbital fibroblasts leading to inflammation and tissue remodelling (3). Recently, a functional crosstalk between TSHR and insulin growth factor-1 receptor (IGF-1R) is known to lead to synergistic stimulation of cellular responses, for example, hyaluronan production in GO orbital fibroblasts (4–6). The orbital volume of both connective tissue and extraocular muscles are increased due to cellular proliferation, inflammation, production of glycosaminoglycan and adipocyte differentiation of orbital fibroblasts (7). B cells, T cells, and orbital fibroblasts play key interactive parts in the pro-inflammatory, pro-fibrotic process (8).. Due to the heterogenic presentation of GO, effective treatment options have been challenging to modify the disease course. Recently advances in the understanding of molecular basis of GO have facilitated research efforts to find new therapeutic targets for the treatment of GO.

Periostin, also known as osteoblast-specific factor 2, is a matricellular protein, which is known to play an important role in extracellular matrix (ECM) structure and organization, particularly in collagen assembly (9). The periostin protein facilitates signaling from the ECM to the cells by binding to integrin α_v_β_3_ receptors, which in turn affects cell adhesion, proliferation, migration, and tissue angiogenesis (10). Current research indicates that periostin is involved in the pathobiology of various diseases including fibrosis, arthritis, atherosclerosis, inflammation, and tumorigenesis (11). Periostin secreted by recruited fibrocytes augments myofibroblast differentiation and lung fibrosis (12). Periostin is also highly secreted from macrophages in visceral adipose tissue in obese mice and periostin deficient mice have lower adipose tissue inflammation (13).

This study was designed to investigate the role of periostin in the pathogenesis of GO. We demonstrated increased periostin expression in GO tissue compared to healthy normal tissue. Knockdown of periostin using siRNA transfection suppressed TGF-β induced profibrotic protein production and IL-1β induced proinflammatory cytokine expression in primary cultured orbital fibroblasts. Our results indicate that periostin may present a potential target to inhibit inflammation as well as the fibrotic process in the mechanism of GO.

## Materials and Method

### Reagents and Chemicals

Antibodies for periostin, β-actin, fibronectin, collagen Iα, α-smooth muscle actin (SMA), phosphorylated (p)- and total (t)- extracellular signal-related kinase (ERK), p-p38, t-p38, p-SMAD 1/5/8, t-SMAD1/5/8, p-SMAD2, t-SMAD2, interleukin (IL)-8, IL-6, monocyte chemoattractant protein-1 (MCP-1), p-nuclear factor (NF)-κB, t-NF-κB, p-Akt, t-Akt, peroxisome proliferator activator gamma (PPARγ), CCAAT-enhancer-binding protein (C/EBP)α and β used in the study are listed in detail in **Supplementary Table 1**. Recombinant TGF-β and IL-1β were obtained form R&D Systems (Minneapolis, UT, USA). Oil Red O was purchased from Sigma-Aldrich, Inc. (Merck KGaA, Darmstadt, Germany). Periostin-targeting siRNA and control siRNA were obtained from Santa Cruz Biotechnology, Inc. (Dallas, TX, USA). TransIT-siQUEST Transfection Reagent was obtained from Mirus Bio, Inc. (Madison, WI, USA). Dulbecco’s modified Eagle’s medium (DMEM), fetal bovine serum (FBS), penicillin, and gentamicin were obtained from Hyclone Laboratories, Inc. (Logan, UT, USA).

### Subjects and Preparation of Tissues and Cells

The GO patient comprised of 8 subjects (age 51.3 ± 14.1 years) as listed in **Supplementary Table 2**. Orbital adipose tissue was obtained during orbital decompression from normally discarded surgical waste. All GO patients had stable euthyroid state for at least 3 months and had not received steroid or radiotherapy treatment for at least 3 months prior to surgery. The control patient population consisted of 8 individuals (age 56 ± 14.8 years) as listed in **Supplementary Table 2**, who underwent either evisceration, orbital wall fracture repair or blepharoplasty. The control patients had no known history or clinical evidence of any thyroid disease. All patients provided written informed consent for the surgical procedure and study participation. The study protocol was reviewed and approved by the Institutional Review Board of Severance Hospital (No. 4-2021-1782), and the study adhered to the tenets of Declaration of Helsinki. The demographics of the subjects of this study are detailed in the **Supplementary Table 2**.

Orbital fibroblasts were cultured by the method used in our previous study (14). Minced tissues were distributed in DMEM:F12 (1:1 ratio) medium containing 20% FBS, penicillin (100 U/ml) and streptomycin (20 mg/ml). The medium was placed in a humidified 5% CO_2_ incubator and kept at 37°C. After confirming the growth of fibroblasts, the cells were serially passaged in monolayers by treating them with trypsin/ethylenediaminetetraacetic acid (EDTA). Strains were stored in liquid nitrogen, and cells were grown in a humidified 5% CO_2_ incubator at 37°C. Cells between the second and fifth passages were used for experiments.

### Quantitative Real Time PCR

To investigate POSTN gene level in GO and healthy orbital tissues, orbital adipose connective tissues were homogenized with a tissue homogenizer (Precellys^®^ 24; Bertin Instruments, Montigeny-le-Bretonneux, France) using a Precellys lysing kit (Bertin Instruments). The RNA was also extracted from cells using Trizol (Thermo Fisher Scientific, Waltham, MA), and the RNA concentration was determined using NanoDrop (Thermo Fisher Scientific). 1µg of mRNA was revere-transcribed into cDNA (Qiagen, Hilden, Germany) and amplified with SYBR green PCR master mix (Takara Bio, Inc., Shiga, Japan) with the QuantStudio 3 Real-Time PCR system (Thermo Fisher Scientific, Waltham MA, USA). The primers sequences for target genes were as follows: *POSTN*; 5’-ACTCAAGATGATTCCCTTT-3’ (forward) and 5’-GGTGCAAAGTAAGTGAAGGA-3’ (reverse), *GAPDH*; 5’-TGCTGTAGCCAAATTCGTTG-3’ (forward) and 5’-CACCCACTCCTCCACCTTT-3’ (reverse). In normalization, GAPDH expression was used. The results as relative changes of fold in the threshold cycle (Ct) value were obtained based on control group, using 2−ΔΔCt method. The experiments were performed three times in cells from a different donor and repeated in duplicate or triplicate per one sample.

### Immunohistochemical Study

Immediately upon surgical removal, both GO and healthy orbital tissues were fixed in 5% formalin and embedded in paraffin after 24 h. Each section was deparaffinized and rehydrated. High-temperature antigen retrieval was achieved by heating the samples in 0.01M citrate buffer for 30 min at full power (750W) in a domestic microwave.The samples were then immersed in methanol containing 0.3% H_2_O_2_ to inactivate endogenous peroxidase at 37°C for 30 min. To remove nonspecific staining, the slides were incubated with appropriate preimmune serum for 30 min at room temperature. After overnight incubation at 4°C with a 1:100 dilution of a primary antibody to periostin (Rabbit polyclonal antibody, Catalog: ab14041, Abcam), slides were rinsed with phosphate-buffered saline (PBS) and incubated with a labeled polymer-HRP was added according to the manufacturer’s instructions and incubated 30 minutes. Color reaction was developed by using 3, 3’-diaminobenzidine tetrachloride (DAB) chromogen solution. All slides were counterstained with hematoxylin. The stained images of each sample were taken using an Olympus BX60 microscope (Olympus, Corp. Melville, NY, USA). For quantification of staining degree, relative values of periostin were estimated by image J program

### Western Blot Assay

Orbital fibroblasts each treated differently by study plan were washed with Dulbecco’s phosphate-buffered saline (DPBS; Welgene, Inc.) and then lysed with RIPA lysis buffer (Welgene, Inc.) containing protease inhibitor cocktail (Thermo Fisher). The cell lysates were resolved in 10% SDS-polyacrylamide gel electrophoresis (SDS-PAGE), and then transferred to nitrocelluose membranes (Millipore Corp., Billerica, MA, USA). The membranes were then treated with primary antibodies overnight at 4°C. Immunoreactive bands were detected with horseradish peroxidase-conjugated secondary antibody with enhanced chemiluminescent substrate (Thermo Fisher) using an image reader (LAS-4000 mini; Fuji Photo Film, Tokyo, Japan). The Intensities of bands, which reflects proteins quantities, were quantified using Image J software (National Institutes of Health, Bethesda, MD, USA) and normalized to that of the β-actin in the same sample.

### Adipogenesis

Adipogenesis of orbital fibroblasts was induced to test the effect of Periostin knockdown on adipogenesis using a previously published protocol (15). Serum-free DMEM supplemented with T3, insulin (Boehringer-Mannheim, Mannheim, Germany), carbaprostaglandin (cPGI2; Calbiochem, La Jolla, CA, USA), and dexamethasone were used for cell culture. A PPARγ agonist, rosiglitazone (10 μM; Cayman, Ann Arbor, MI, USA), was also added from day 1 of differentiation to enhance stimulation of adipogenesis. To evaluate the effect of silencing periostin on adipogenesis, cells were transfected with control-siRNA or periostin-targeting siRNA for 24h before 14-day differentiation period.

### Oil Red O Staining of Cells

Cells were stained with Oil Red O as described by Green and Kehinde (16). Briefly, six milliliters of a stock solution prepared with 0.5% Oil Red O in isopropanol was mixed with 4 ml of distilled water and placed at room temperature for 1 hour. Then the solution was filtered,added to cells washed with PBS, and fixed with 3.7% formalin at 4°C for 1 hour. The cell-Oil Red O solution mixture was left at room temperature for 1 hour and examined using a light microscope (Axiovert; Carl Zeiss AG, Oberkochen Germany) and photographed (Olympus BX60; Olympus Corp., Melville, NY, USA).

### Statistical Analysis

For statistical analyses, IBM SPSS statistic for Window v 20.0 (IBM Corp., Armonk, NY, USA) was used. Experiments were repeated a minimum of three times with each patients’ samples. The Mann-Whitney U-test and Kruskal-Wallis test were used for nonparametric data, and the Kolmogorov-Smirnov test was used for data that was not normally distributed. A p-value less than 0.05 was considered statistically significant.

## Results

### GO Tissues Show Increased *POSTN* Gene Expression

**Figure 1:**
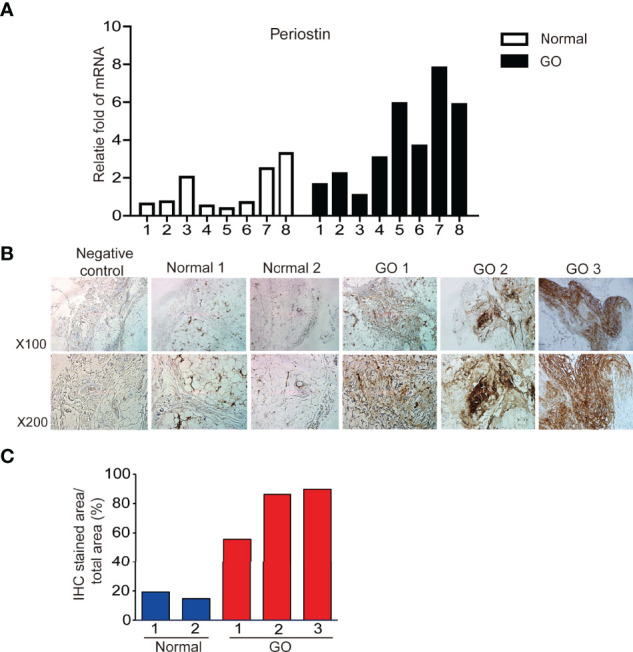
Expression of periostin gene and protein between GO and normal tissues. Expression of *POSTN* gene expression **(A)** in GO (n=8) and normal healthy tissues (n=8) was analyzed and compared using real time PCR. Immunohistochemical staining for periostin in normal and GO tissues were carried out **(B)**. Stronger staining for periostin was seen in the GO tissues than in normal tissue **(C)**.

*POSTN* gene expression was quantified using real time PCR and compared between orbital tissues from GO (n=8) and normal healthy (n=8) subjects. *POSTN* transcript levels were expressed significantly higher in GO tissues than normal tissues (**Figure 1A**). Immunohistochemical staining of orbital tissues revealed that periostin was overexpressed in GO tissues (n=3), but only faint staining was found in normal healthy orbital tissue (n=2) (**Figure 1B**). By quantitative analysis of the immunostaining results, periostin expression was increased 2.5–4.5 fold in GO tissues, compared to that in normal healthy tissue (**Figure 1C**).

**Figure 2:**
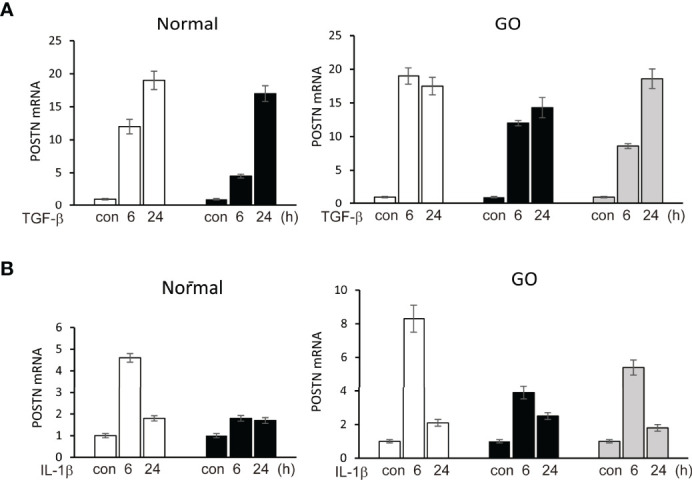
Upregulation of *POSTN* gene by stimulation of TGF-β and IL-1β. **(A)** TGF-β (5 ng/ml) upregulated expression of *POSTN* gene in a time-dependent manner in both normal (n=2) and GO (n=3) cultured orbital fibroblasts in real time PCR analyses. **(B)** *POSTN* gene expression was increased when cells were treated with 10 ng/ml of IL-1β for 6 h in normal (n=1) and GO (n=3) cultured orbital fibroblasts. The folds for the mRNA values are shown for RT-PCR.

In primary cultured orbital fibroblasts, *POSTN* gene expression was significantly increased after stimulation of TGF-β (5 ng/ml) for 6 and 24 hours in both GO (n=3) and normal (n=2) orbital fibroblasts in a time-dependent manner in real time PCR analyses (**Figure 2**). IL-1β (10 ng/ml) at 6 h of treatment upregulated POSTN gene expression in GO (n=3) and normal (n=1) orbital fibroblasts.

### Knockdown of Periostin Suppresses TGF-β-Induced Profibrotic Proteins Production

**Figure 3:**
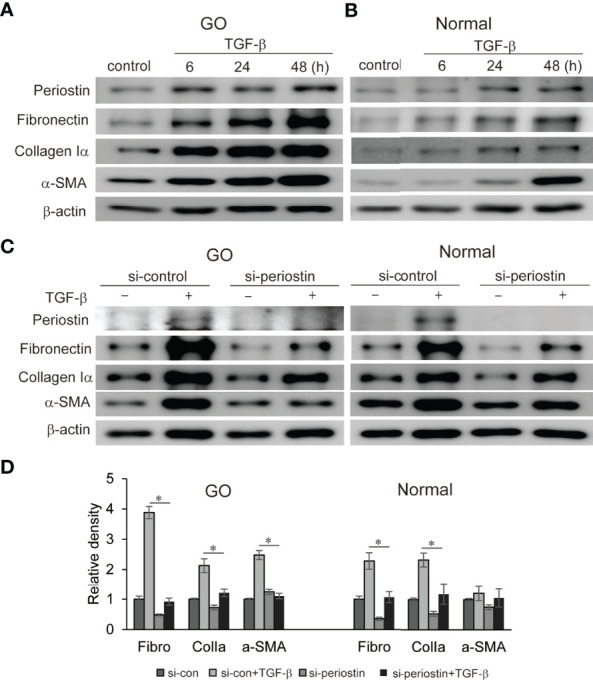
Effect of silencing periostin on production of TGF-β-induced profibrotic proteins in GO and normal fibroblast. **(A, B)** Orbital fibroblasts from GO patients (n=3) and normal patients (n=3) were treated with 5 ng/ml TGF-β for 6-48 hours. Production of periostin was increased in a time dependent manner, more predominantly in GO orbital fibroblasts. **(C, D)** Knockdown of periostin by siRNA transfection ameliorated TGF-β-induced production of profibrotic protein in Western blot analyses. Fibronectin (Fibro) and collagen Iα (Colla) expression was suppressed in both GO and normal cells, and α-SMA expression was inhibited in GO cells. The representative gel images are also shown, and the results from orbital fibroblasts are presented as the mean density ratio ± SD, normalized to the level of β-actin in the same sample. (*p < 0.05 vs. TGF-β-stimulated cells).

Fibrosis in GO has a profound impact on eye motility which has a negative impact on quality of life. TGF-β, a major mediator of chronic fibrosis is secreted by various cells including macrophages and fibroblasts (Ludgate, 2020). We found that TGF-β significantly induces periostin protein production in a time-dependent manner in both GO (n=3) and normal (n=3) orbital fibroblasts in western blot analysis (**Figures 3A, B**). In addition, TGF-β (5ng/ml) induces profibrotic protein expression such as fibronectin, collagen Iα, and α-SMA in a time-dependent manner (**Figures 3A, B**). When the periostin gene was silenced using siRNA (100 nM, 24 h) before TGF-β stimulation, enhanced expression of fibronectin, collagen Iα and α-SMA were significantly suppressed in both GO and normal orbital fibroblasts (**Figure 3C, D**).

### Effect of Silencing *POSTN* Gene on Activation of Canonical and Noncanonical TGF-β Signaling Pathway

**Figure 4:**
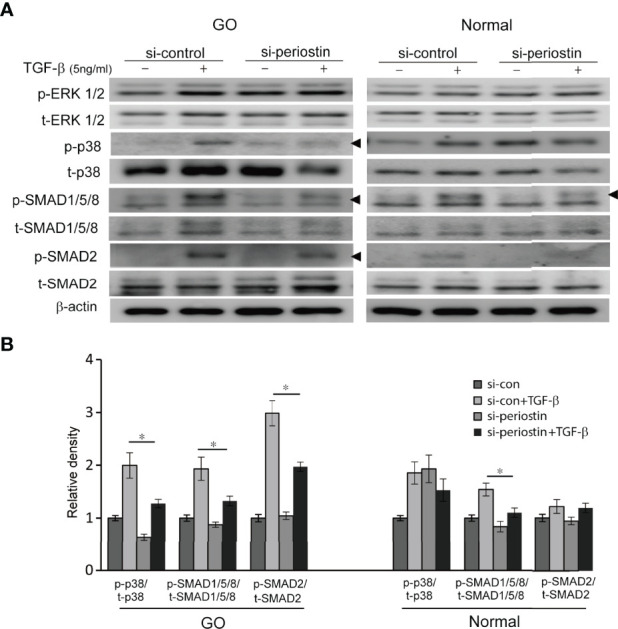
Effect of silencing periostin on the activation of signaling molecules in response to TGF-β treatment. **(A)** Orbital fibroblasts from GO patients (n=3) and normal patients (n=3) were treated with 5 ng/ml TGF-β for 15 minutes after cells were transfected with control siRNA or periostin-targeting siRNA for 48 h. **(B)** Knockdown of periostin suppressed TGF-β induced phosphorylation of p38, SMAD1/5/8 and SMAD 2 protein in GO orbital fibroblasts. Phosphorylation of SMAD1/5/8 protein was inhibited by silencing periostin in normal orbital fibroblast. The representative gel images are also shown, and the results from orbital fibroblasts are presented as the mean density ratio ± SD, normalized to the level of β-actin in the same sample. (*p < 0.05 vs. TGF-β-stimulated cells).

To investigate the effect of silencing periostin on TGF-β downstream signal pathway, the expression of p- and t- MAPK signaling molecule and SMAD protein was analysed by western blot. In orbital fibroblasts from GO, activation p38, SMAD1/5/8 and SMAD2 protein by TGF- β treatment (5 ng/ml, 1h) was ameliorated by periostin knockdown (**Figure 4**). Only SMAD1/5/8 protein expression was inhibited by silencing of periostin in normal orbital fibroblasts.

### Knockdown of Periostin Suppresses IL-1β-Induced Proinflammatory Cytokine Production

**Figure 5:**
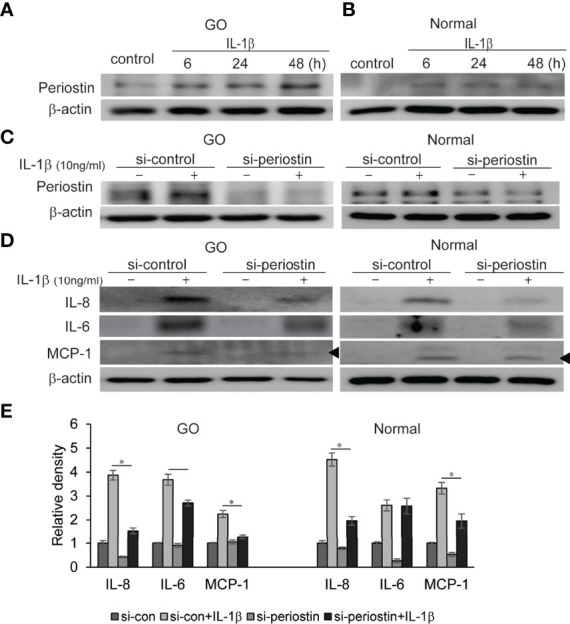
Effect of silencing periostin on production of IL-1β-induced proinflammatory proteins in GO and normal fibroblast. Orbital fibroblasts from GO patients (n=3) **(A)** and normal patients (n=3) **(B)** were treated with 10 ng/ml IL-1β for 6-48 hours. IL-1β increased expression of periostin protein in a time-dependent manner in GO cells, more predominantly. **(C, D)** Knockdown of periostin by siRNA transfection ameliorated IL-1β -induced production of proinflammatory protein in Western blot analyses. IL-1β induced IL-6, IL-8 and MCP-1 protein production was suppressed by silencing periostin in both GO and normal orbital fibroblasts. Representative bands from western blot analyses were shown **(A–D)**. **(E)** The results from orbital fibroblasts are presented as the mean density ratio ± SD, normalized to the level of β-actin in the same sample. (*p < 0.05 vs. IL-1β-stimulated cells).

IL-1β plays a critical role of cytokine and chemokine production involved in inflammatory autoimmune pathogenesis of GO. We demonstrate that IL-1β (10 ng/ml) remarkably enhanced production of periostin protein in a time-dependent manner in western blot analysis (**Figures 5A, B**). Periostin was silenced using siRNA transfection in both GO and normal orbital fibroblasts (100 nM, 24 h) before IL-1β stimulation. The production of proinflammatory cytokines including IL-8, IL-6 and MCP-1 protein induced by IL-1β treatment (10 ng/ml, 48 h) was attenuated by periostin knockdown in GO cells (**Figures 5C–E**). Similarly, IL-1β induced IL-8 and MCP-1 protein expression was suppressed by silencing periostin in normal orbital fibroblasts.

### Effect of Silencing Periostin on Activation of Proinflammatory Signaling Molecules

**Figure 6:**
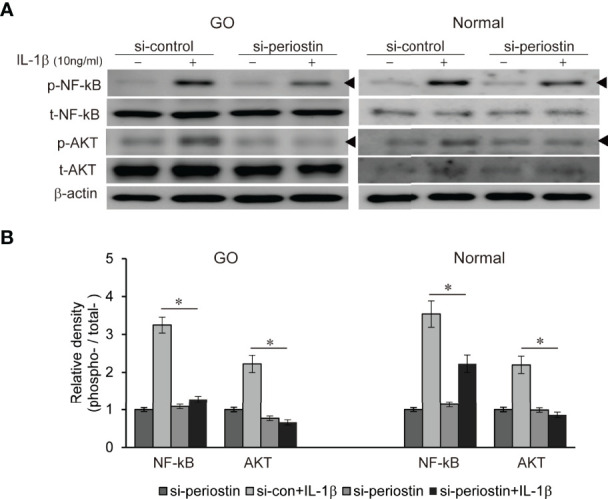
Effect of silencing periostin on production of IL-1β-induced signalling molecule in GO and normal fibroblast. Orbital fibroblasts from GO patients (n=3) and normal patients (n=3) were treated with 10 ng/ml IL-1β before cells were transfected with control siRNA or periostin-targeting siRNA. Knock down of periostin attenuated IL-1β-induced increase of phosphorylated NF-kB and AKT protein in both GO and normal orbital fibroblasts. **(A)** Representative bands from western blot analyses were shown **(A)**, and **(B)** the results are presented as the mean density ratio ± SD, normalized to the level of β-actin in the same sample. (*p < 0.05 vs. IL-1β -stimulated cells).

Both TSH receptor and IGF-1 receptor form a complex to drive a signaling *via* phosphatidyl inositol 3-kinase (PI3K)/Akt/mTOR pathways leading to pathogenesis of GO (17). We previously found PI3K delta inhibitor, idelalisib blocks inflammatory pathogenesis of GO in orbital fibroblast (18). NF-kB is well-known proinflammatory mediator, and plays a crucial role in the maintenance of inflammatory condition in autoimmune disease (19). Periostin was silenced using siRNA transfection (100 nM, 24 h) in both GO and normal orbital fibroblasts before IL-1β treatment (10ng/ml, 1 h) (**Figure 6**). Increased p-NFκB and p-Akt protein under IL-1β stimulation was substantially suppressed by periostin knockdown in both GO and normal orbital fibroblasts.

### Silencing Periostin Suppresses Adipocyte Differentiation in GO Orbital Fibroblasts

**Figure 7:**
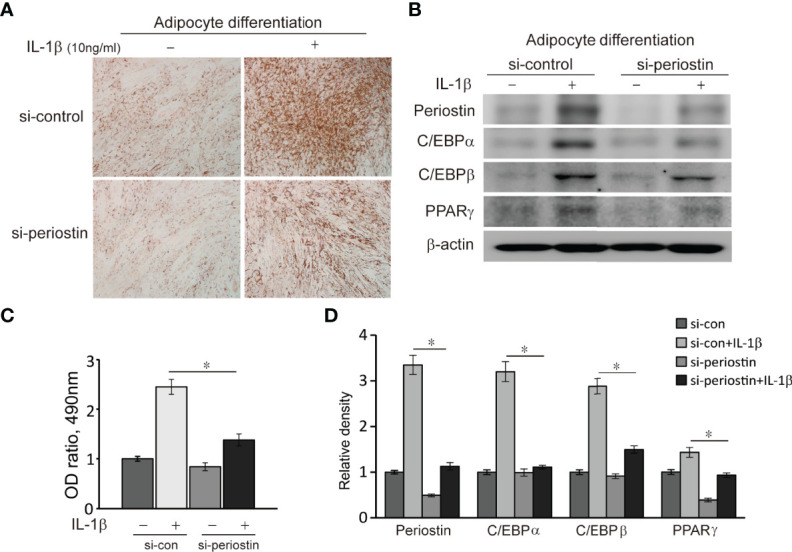
Effect of silencing periostin in adipogenesis of Graves’ orbital fibroblasts. Cells from GO patients were grown in adipogenic media for 14 days after cells were transfected using si-control or si-periostin RNA transfection. IL-1β (10 ng/ml) was added to the adipogenic medium to further stimulate differentiation of fibroblasts into adipocyte. At 14 days of adipogenesis, cells were stained with Oil Red O and examined using a microscope at 40X, and western blot analyses were performed to find the effect of silencing periostin on adipogenesis. **(A, C)** Periostin knock down significantly reduced oil red O-stained cells and **(B, D)** adipogenic transcriptional regulators, C/EBPα, C/EBPβ and PPARγ protein production, especially in IL-1β stimulated condition. Experiments were conducted on 3 different GO cell strains, and the representative microscopic and gel images are shown. (*p<0.05 vs. IL-1β-stimulated cells).

Orbital fibroblasts from GO patients were cultured in the adipogenic medium for 14 days to induce adipogenic differentiation. The orbital fibroblasts lost their stellate original appearance and morphologically transformed into a spherical shape with the accumulation of intracellular lipid droplets. The differentiated cells significantly increased when IL-1β was added to the adipogenic medium. Transfection of differentiating fibroblasts with periostin-siRNA substantially attenuated adipogenesis as identified with Oil Red O staining on day 14 (**Figure 7**). Periostin inhibition substantially attenuated levels of adipogenic transcription factors, PPARγ, C/EBPα and β protein in western blot analyses (**Figure 7**).

## Discussion

In this study, we identified higher expression of *POSTN* gene in GO tissues compared to normal healthy orbital tissue, which was significantly upregulated by TGF-β or IL-1β stimulation in primary cultured orbital fibroblasts. We present that inhibition of periostin using siRNA transfection suppresses the production of TGF-β induced profibrotic protein by blocking both canonical and noncanonical signal pathways of TGF-β. In addition, periostin knockdown suppresses IL-1β induced proinflammatory cytokine production by inhibiting phosphorylation of NF-kB and Akt signaling protein. Silencing periostin also suppresses adipocyte differentiation of orbital fibroblasts from GO. Based on our results, periostin may play a significant role in the main pathogenesis of GO including fibrosis, inflammation, and adipogenesis.

Periostin has been suggested to function as a scaffold for assembly of several ECM proteins due to its multi-domain structure (20) and controls collagen formation with effective collagen cross-linking (21). Periostin promotes inflammation and fibrosis in different disease states including myocardial infarction, idiopathic pulmonary fibrosis, asthma, skin scleroderma, hepatic fibrosis, muscular dystrophy and chronic renal disease (22). In areas of ongoing fibrosis in lung tissues from idiopathic pulmonary fibrosis patients or in the lungs of asthmatic patienta, periostin is highly expressed, contributing to airway fibrosis and remodeling (23). Serum levels of periostin in idiopathic pulmonary fibrosis are significantly high, correlating with the disease severity, suggesting periostin as a predictive biomarker of lung fibrosis (24, 25). In chronic fibrotic kidney disease, periostin levels in the fibrotic tissue and urine were highly related to the pathologic stage and the decline of renal function (26). Genetic or pharmacologic inhibition of periostin blocked the progression of renal fibrosis, providing a novel biomarker as well as target of therapy for chronic kidney disease (27). There have been numerous reports that the crosstalk between TGF-β and periostin leads to pulmonary fibrosis and inhibitors for integrin α_v_β_3_, a periostin receptor can block pulmonary fibrosis in animal model and TGF-β signaling in lung fibrosis (28).

GO patients exhibit enlarged orbital fat volume as well as extraocular muscles. Accumulation of hydrophilic hyaluronan with tissue edema also results in soft tissue volume expansion. Cell proliferation, adipocyte differentiation elicited by immune cell infiltration within orbit, is also responsible for tissue expansion. Although GO usually resolves spontaneously to some extent, chronic fibrosis of tissues might remain causing motility restriction and proptosis unresponsive to steroid treatment. TGF-β, a potent cytokine secreted by macrophage and fibroblasts plays a central role in promoting fibrosis in the orbit, however to date, no effective treatments have emerged to block TGF-beta inducing fibrotic pathology with excess deposition of ECM, although there are several encouraging results of anti-fibrotic agents in preclinical and early clinical studies (29). There remains an unmet need to develop a novel therapeutic drug to halt chronic progression of GO. We have found that silencing periostin suppresses TGF-β induced profibrotic phenotype as well as canonical and noncanonical TGF-β signaling pathway. Of the noncanonical signal pathway, activated phosphorylation of p38 protein by TGF-β stimulation was attenuated by periostin inhibition. We believe our *in vitro* data suggests periostin as a novel anti-fibrotic target of GO.

Periostin is highly expressed in chronic inflammatory diseases, including asthma, atopic dermatitis, chronic rhinosinusitis with nasal polyp, and allergic conjunctivitis, suggesting that periostin plays important roles in the pathogenesis of inflammatory diseases (30). Type 2 immunity cytokines such as IL-4 and IL-13 stimulates expression of periostin, promoting chronic allergic reaction (31, 32). Periostin is also upregulated in other chronic inflammations such as inflammatory bowel disease and rheumatoid arthritis (33, 34). Silencing of periostin attenuates IL-8 mRNA expression and NF-kB DNA-binding activity in intestinal epithelial cells (33). Mice lacking the periostin gene show less interstitial inflammation as well as fibrosis preventing structural alterations in ureteral obstruction model (35). Periostin expression is increased in mechanically strained vascular smooth muscle cells, followed by MCP-1 secretion, which are blocked by a periostin-neutralizing antibody (36). Periostin promoter activity is highly induced by a key proinflammatory transcriptional factor, p65 NF-kB subunit in the nephrotoxic serum nephritis model, and recombinant periostin treatment in cultured podocyte increases its receptor integrin-β_3_ expression followed by AKT activation resulting in pathologic phenotype change (37). Periostin has also been shown to activate NF-kB and enhance mTOR (30). NF-kB and AKT signal protein are major key players of the inflammatory pathogenesis of GO, and their function in CD40-mediated signaling pathway of IL-6 production in fibrocyte in GO has been reported (38). In our results, lack of periostin expression significantly reduces proinflammatory cytokine production including IL-6, IL-8 and MCP-1 and suppresses activation of NF-kB and AKT signal protein, suggesting a novel potential therapeutic target of inflammatory mechanism of GO.

In this study we demonstrate that a periostin knockdown suppresses adipocyte differentiation and related transcriptional factor protein production in GO orbital fibroblasts, possibly by attenuating cell proliferation. During adipocyte differentiation in GO, the transition between cell proliferation and differentiation takes place (39), and simultaneously cellular proliferation of orbital fibroblasts are triggered by complex molecular pathways. Autologous T cells stimulate proliferation of orbital fibroblasts (40), and GO cells show enhanced proliferative capacity at baseline and in response to certain cytokines compared to controls (41). Periostin is an active molecule in tumor microenvironments promoting cancer progression through various mechanism including proliferation, invasion and angiogenesis (42). Its expression is upregulated in various cancers including non-small cell lung cancer (43), prostate cancer (44), renal cell carcinoma (45) and breast cancer (46). Recombinant periostin induces a significant increase in the proliferation of hypertrophic scar fibroblasts but not in normal dermal fibroblasts (47). Furthermore, knockdown of periostin suppresses cigarette smoke extract induced proliferation of pulmonary arterial smooth muscle cells, which is significantly enhanced by recombinant periostin n (48). Oxidative stress, such as cigarette smoke extract or hydrogen peroxide, stimulates adipocyte differentiation with increased generation of reactive oxygen species in orbital fibroblasts, which is reversed by an antioxidant (49). Periostin could be induced in proliferating and differentiating cells under stressed conditions and with periostin blockage alleviating adipogenesis possibly *via* anti-proliferative effect, although the exact mechanism is unclear.

Since we aimed to investigate the role of periostin in *an in vitro* model of GO, thus we did not investigate serum periostin levels in the subjects at this time. To evaluate clinical relevance of periostin in GO, analyzing serum periostin level of blood samples according to clinical inflammatory activity or severity could be helpful and act as a bridge to potential future clinical application of periostin. Periostin has potential to become a novel prognostic biomarker and treatment target for many diseases including chronic fibrosis, inflammation, allergy and cancer. Enhanced periostin levels can be detected in either tissue biopsy samples or serum, as periostin is secreted from pathologic tissue and transported into blood vessels (50–52). Several preclinical and clinical trials have been applied to demonstrate the efficacy of blocking periostin. An anti-periostin peptide was synthesized and proved to reverse resistance to doxorubicin in breast cancer cells (53). Inhibiting periostin using antibody binding α_v_β_3_ integrin region has been developed and proved effective in cancer cells, however *in vivo* successful is still pending. Recently, a periostin-binding aptamer has been developed with improved affinity and specificity compared to antibodies or small molecules. Intraperitoneal administration of a periostin aptamer significantly inhibited peritoneal fibrosis (54), and blunted profibrotic protein production and blood urea nitrogen level in diabetic mice (55).

In conclusion, our study showed for the first time that periostin is overexpressed in GO tissues and inhibition of periostin reverses the pathologic GO phenotypes of fibrosis, inflammation and adipogenesis. The precise role of periostin during GO disease progression is still unclear, however our data suggests that periostin might act as a key mediator of GO pathogenesis, providing *in vitro* evidence that targeting periostin for treatment could be a novel modality to alleviate chronic progression of disease. Nonetheless, an *in vivo* confirmatory study is mandatory to explore the possibility of clinical translation.

## Data Availability Statement

The raw data supporting the conclusions of this article will be made available by the authors, without undue reservation.

## Ethics Statement

The study protocol was reviewed and approved by the Institutional Review Board of Severance Hospital (No. 4-2021-1782). The patients/participants provided their written informed consent to participate in this study.

## Author Contributions

SJ, CL, and JY wrote the first draft of the manuscript and formal analysis. JK, JP, CL, and BK investigated. JY contributed to conception. DK, EL, and JY review and edited the manuscript. All authors contributed to manuscript revision, read, and approved the submitted version.

## Funding

This work was also supported by the National Research Foundation of Korea Grant funded by the Korean Government (NRF-2021R1F1A1046652), and by a National Research Foundation of Korea (NRF) grant funded by the government of Korea (MSIT) (No. 2020R1A2C4002095), and was partially supported by the Soonchunhyang University Research Fund.

## Conflict of Interest

The authors declare that the research was conducted in the absence of any commercial or financial relationships that could be construed as a potential conflict of interest.

## Publisher’s Note

All claims expressed in this article are solely those of the authors and do not necessarily represent those of their affiliated organizations, or those of the publisher, the editors and the reviewers. Any product that may be evaluated in this article, or claim that may be made by its manufacturer, is not guaranteed or endorsed by the publisher.
