# Supplementary material for: Therapeutic Potential of Targeting Periostin in the Treatment of Graves’ Orbitopathy
Source: Front Endocrinol (Lausanne). 2022 May 30;13:900791. doi: 10.3389/fendo.2022.900791 (PMC9189304; doi:10.3389/fendo.2022.900791)
Supplement: Supplementary file 1 [file Table_1.docx]

**Supplementary table 1.** List of reagents.

| Antibody^*^ | Manufacturer | Catalogue number | Species |
| --- | --- | --- | --- |
| Periostin | Abcam | ab14041 | Human, Mouse, Rat |
| β-actin | Santa Cruz Biotechnology | sc-47778 | Human, Mouse, Rat |
| Fibronectin | BD | 610077 | Human, Mouse, Rat |
| Collagen Ia | Abcam | ab 90395 | Human, Rabbit, Bovine |
| α-SMA | Sigma | A5228 | Human, Mouse, Rat |
| p-ERK | Cell Signaling Technology | 4376 | Human, Mouse, Rat |
| t-ERK | Cell Signaling Technology | 9102 | Human, Mouse, Rat |
| p-p38 | Cell Signaling Technology | 9211 | Human, Mouse, Rat |
| t-p38 | Cell Signaling Technology | 9212 | Human, Mouse, Rat |
| p-SMAD1/5/8 | Cell signaling | 13820 | Human, Mouse, Rat |
| t-SMAD1 | Cell signaling | 9743 | Human, Mouse, Monkey |
| p-SMAD2 | Cell signaling | 3108 | Human, Mouse, Rat |
| t-SMAD2 | Cell signaling | 3102 | Human, Mouse, Rat |
| IL-8 | Abcam | ab7747 | Human |
| IL-6 | Novus Biologicals | NB600-1131 | Human, Mouse, Rat |
| MCP-1 | Cell Signaling Technology | 2027 | Human |
| p-p65 NFκB | Cell Signaling Technology | 3031 | Human, Mouse, Rat |
| p65 NFκB | Cell Signaling Technology | 4764 | Human, Mouse, Rat |
| p-Akt | Cell Signaling Technology | 9271 | Human, Mouse, Rat |
| t-Akt | Cell Signaling Technology | 9272 | Human, Mouse, Rat |
| PPARγ | Santa Cruz Biotechnology | sc-7273 | Human, Mouse, Rat |
| C/EBPα | Santa Cruz Biotechnology | sc-61 | Human, Mouse, Rat |
| C/EBPβ | Santa Cruz Biotechnology | sc-7962 | Human, Mouse, Rat |

Abbreviations: IL**,** Interleukin; MCP-1, Monocyte chemoattractant protein-1; PPARγ, Peroxisome proliferator activator gamma; C/EBP, CCAAT-enhancer-binding protein; p-, phosphorylated; t, total; SMAD, Suppressor of mothers against Decapentaplegic*;* NF-κB, Nuclear factor kappa-light-chain-enhancer of activated B; ERK, Extracellular signal-regulated kinase; α-SMA, α-Smooth muscle actin

^*^The antibodies are listed in the order of appearance.

**Supplementary table 2.** Clinical information of patient samples used for all *in vitro* studies

| Age (years) | sex | CAS | Smoker | Duration of GO (years) | Proptosis R/L (mm) | | Surgery performed |
| --- | --- | --- | --- | --- | --- | --- | --- |
| GO patients (n=8) | | | | | | | |
| 44 | F | 0/7 | n | 11 | 21/21 | Decompression | |
| 61 | F | 1/7 | n | 2 | 23/23 | Decompression | |
| 45 | F | 0/7 | y | 3 | 26/26 | Decompression | |
| 69 | M | 1/7 | y | 1.8 | 19/19 | Decompression | |
| 41 | F | 0/7 | n | 8 | 22/22 | Decompression | |
| 31 | M | 0/7 | y | 4 | 23.5/23.5 | Decompression | |
| 54 | M | 4/7 | y | 0.5 | 18/19 | Decompression | |
| 69 | M | 2/7 | y | 2.2 | 21/21 | Decompression | |
| Normal control subjects (n=8) | | | | | | | |
| 33 | M | n/a | n | n/a | n/a | | Evisceration |
| 75 | M | n/a | n | n/a | n/a | | Evisceration |
| 35 | F | n/a | y | n/a | n/a | | Orbital wall fracture |
| 56 | F | n/a | n | n/a | n/a | | Upper blepharoplasty |
| 66 | F | n/a | n | n/a | n/a | | Upper blepharoplasty |
| 65 | M | n/a | n | n/a | n/a | | Upper blepharoplasty |
| 57 | M | n/a | n | n/a | n/a | | Lower blepharoplasty |
| 61 | F | n/a | n | n/a | n/a | | Lower blepharoplasty |

GO, Graves’ orbitopathy; CAS, clinical activity scores; Y, yes; N, No; N/A, not applicable; F, female; M, male; R, right eye; L, left eye.
