# Supplementary figures and images for: Therapeutic Potential of Targeting Periostin in the Treatment of Graves’ Orbitopathy
Source: Front Endocrinol (Lausanne). 2022 May 30;13:900791. doi: 10.3389/fendo.2022.900791 (PMC9189304; doi:10.3389/fendo.2022.900791)

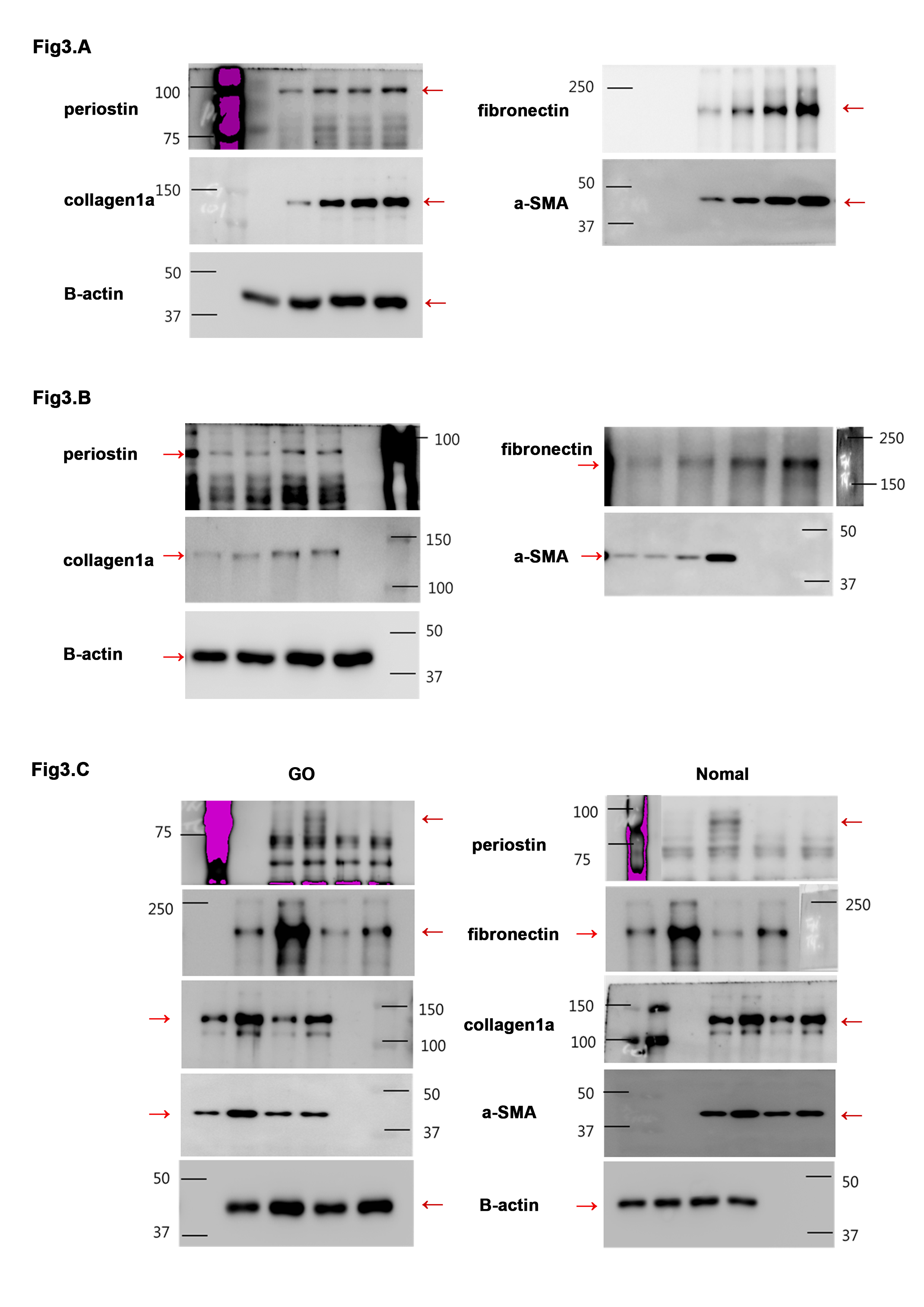

Supplement: Supplementary file 2 [file Image_1.tif]

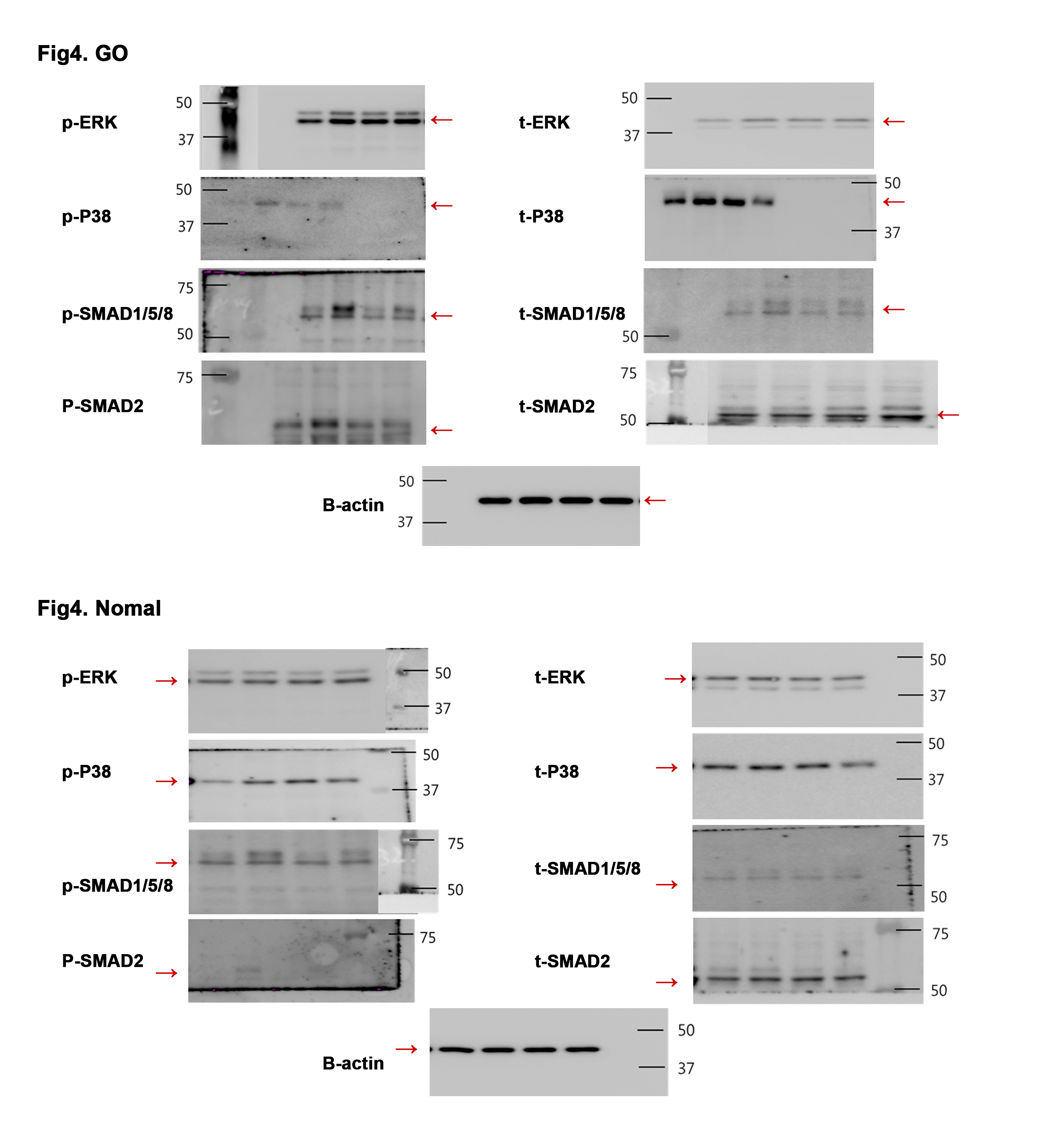

Supplement: Supplementary file 3 [file Image_2.tif]

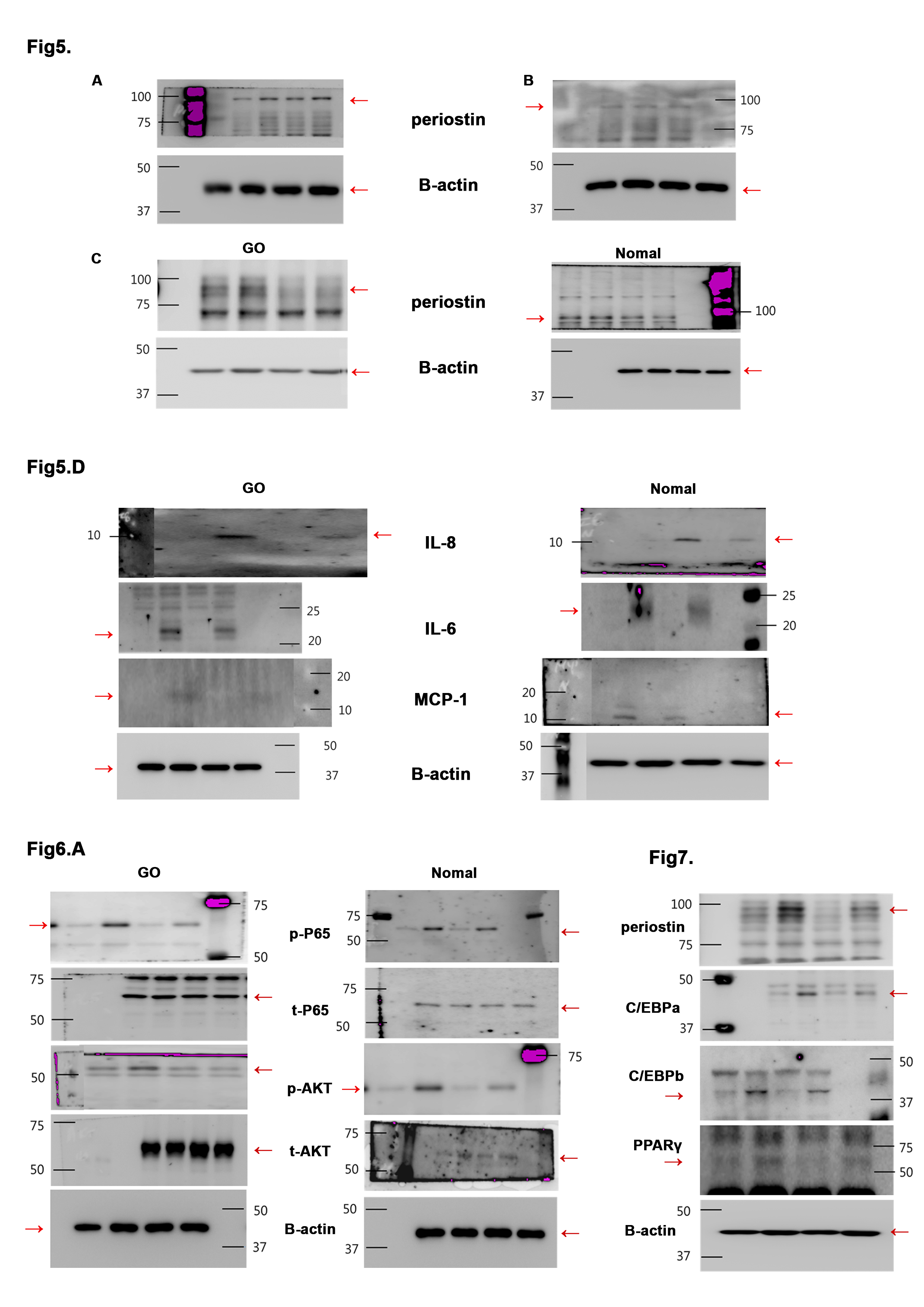

Supplement: Supplementary file 4 [file Image_3.tif]
